# Micronutrient Status among Pregnant Women in Zinder, Niger and Risk Factors Associated with Deficiency

**DOI:** 10.3390/nu9050430

**Published:** 2017-04-26

**Authors:** K. Ryan Wessells, Césaire T. Ouédraogo, Rebecca R. Young, M. Thierno Faye, Alex Brito, Sonja Y. Hess

**Affiliations:** 1 Program in International and Community Nutrition, Department of Nutrition, University of California, Davis, CA 95616, USA; ctouedraogo@ucdavis.edu (C.T.O.); rryoung@ucdavis.edu (R.R.Y.); syhess@ucdavis.edu (S.Y.H.); 2 Helen Keller International, Niamey 0000, Niger; tfaye@hki.org; 3 USDA, ARS, Western Human Nutrition Research Center, Davis, CA 95616, USA; abrito@ucdavis.edu

**Keywords:** micronutrient, deficiency, anemia, pregnancy, antenatal care, iron, zinc, vitamin

## Abstract

Anemia and micronutrient (MN) deficiencies in pregnant women are associated with adverse pregnancy outcomes. In Niger, 58.6% of pregnant women are anemic; however, MN statuses are unknown. The study objectives were to estimate the prevalence of MN deficiencies among pregnant women in Zinder, Niger and explore associated risk factors. Pregnant women living in randomly selected rural villages (*n* = 88) were included. Capillary and venous blood samples (*n* = 770) were analyzed for hemoglobin (Hb) and plasma ferritin, soluble transferrin receptor (sTfR), zinc (pZn), retinol binding protein (RBP), folate and vitamin B_12_. C-reactive protein and alpha-1-acid glycoprotein were measured to adjust for inflammation. The prevalence of MN deficiencies in pregnant woman was high, indicative of a severe public health problem. Prevalence of iron deficiency was 20.7% and 35.7%, by ferritin (<15 µg/L) and sTfR (>8.3 mg/L), respectively. In total, 40.7% of women had low pZn (<50 µg/dL), 79.7% had marginal RBP (<1.32 µmol/L), 44.3% of women had low folate (<10 nmol/L) and 34.8% had low B_12_ concentrations (<148 pmol/L). Common risk factors associated with MN status included gravidity, mid-upper-arm circumference, geophagy, malaria, and result of the woman’s last pregnancy. Interventions to promote the strengthening of antenatal care, and access and adherence to nutrition and health interventions are critical among pregnant women in this population.

## 1. Introduction

Micronutrient (MN) deficiencies in pregnancy are associated with adverse health outcomes for both the pregnant woman and her offspring, including maternal anemia, maternal and perinatal mortality, and low birth weight, pre-term birth, intra-uterine growth restriction, altered immune response and cognitive deficits in the newborn [1,2,3,4,5,6,7].

Physiological MN requirements increase during pregnancy to meet increased maternal metabolic demands, increased erythropoiesis, accretion of maternal tissue reserves and fetal requirements for growth and development. Women in low-income countries are particularly vulnerable to MN deficiencies during pregnancy, owing to beginning pregnancy with MN deficiencies or depleted stores, inadequate dietary intakes during pregnancy (e.g., limited access to and consumption of foods rich in micronutrients and poor absorption), frequent exposure to infection, initiation of pregnancy during adolescence, high fertility rates and short inter-pregnancy intervals [4,8]. 

In Niger, the fertility rate (7.6) and the adolescent birth rate (204.8 births per 1000 women) are the highest in the world [9,10]. The maternal mortality ratio is also high at 535 deaths per 100,000 live births [9]. MN deficiencies among pregnant women in Niger are believed to be a serious problem, but there is only limited information available on women’s nutritional status during pregnancy. In 2012, 58.6% of pregnant women in Niger were anemic [9]; in Western sub-Saharan Africa, iron deficiency anemia among women is the top ranking cause of anemia in the region [11]. In addition, the proportion of pregnant women with night blindness was previously estimated at 6.6% [12] and 15.5% of women of child-bearing age are undernourished (BMI < 18.5 kg/m^2^) [9]. 

Current World Health Organization (WHO) guidelines recommend daily supplementation with iron (60 mg elemental iron) and folic acid (400 µg) for women during pregnancy in settings where anemia during pregnancy is a severe public health problem (>40%) [13]. In Niger, iron and folic acid (IFA) supplements are almost exclusively available to pregnant women through antenatal care (ANC) at governmental health clinics, and policies issued by the Ministry of Public Health are in line with WHO guidelines [14]. However, a previous survey of nutrition services provided during ANC revealed that health care providers’ adherence to guidelines is inadequate, and knowledge transfer to pregnant women is limited [15]. In observed ANC consultations, only 57% of pregnant women received IFA supplements, and 38% received counseling on pregnancy-related dietary improvements [15]. In the 2012 Demographic and Health Survey (DHS), 81% of women reported IFA consumption at any point during their last pregnancy, yet only 29% of women consumed supplements for at least 90 days [9]. For the prevention and control of anemia due to infection, 20% of pregnant women report sleeping under an insecticide treated mosquito net, 35% reported receiving intermittent preventive treatment of malaria in pregnancy, and 52% reported receiving antihelminthics [9]. The primary source for nutrition and health status of pregnant women in Niger is the 2012 DHS and limited information is available on the prevalence and risk factors of MN deficiencies in Niger. Therefore, the objectives of this study were: (1) to assess MN status of pregnant women in Zinder, Niger; and (2) to identify risk factors associated with MN status and high-risk population sub-groups that could be targeted for more intensive ANC and public health interventions.

## 2. Materials and Methods

### 2.1. Study Design

This study was a community-based cross-sectional survey among pregnant women in rural communities in the Zinder Region of Niger, and was conducted as a part of the baseline assessment for the Niger Maternal Nutrition (NiMaNu) Project. The NiMaNu project was a programmatic intervention to improve ANC services, designed with a rigorous research component to assess and evaluate the impact of the intervention. The project was implemented March 2014–September 2015, using a continuous enrollment schedule over a period of 18 months to account for potential effects of seasonality in the outcome measures.

### 2.2. Study Participants

Pregnant women in rural villages within the catchment areas of 18 out of 45 governmental integrated health centers (CSI) located in two districts (Zinder and Mirriah) of the Zinder Region of Niger were eligible to participate. CSI were selected based on convenience sampling and randomized to the order of participation; criteria for inclusion were determined in collaboration with local governmental health officials and included year-around accessibility, distance to regional capital (Zinder) and a limited number of interventions being implemented in the catchment area. Within each catchment area of each CSI, the village in which the CSI was located was automatically included in the study. In addition, one village with a health post (CS) was randomly selected and included. Finally, from among the remaining villages in the catchment area of the CSI, four communities ≤10 km from the CSI and four communities >10 km from the CSI were randomly selected and randomized to order of participation. Women from the first 4 villages in each CSI catchment area (CSI-village, CS-village, and 2 additional villages) were enrolled with a target of approximately 16–20 women enrolled per village. If the sample size of approximately 77 women per CSI catchment area was not met by the first 4 randomized villages of each CSI, then additional villages on the randomization list of each CSI catchment area were included until the sample size was reached. A total of 88 communities were included in the present survey. Pregnant women were identified using the random walk method [16], with the starting point randomly selected for each village (market, primary school or mosque). 

Pregnant women at any week of gestation were eligible to participate in the survey if they provided written informed consent, had resided in the village for the previous six months and had no plans to move out of the study area within the coming two months. Women were ineligible if they had an illness warranting immediate hospital referral or were unable to provide consent due to mental disability. 

### 2.3. Ethical Considerations

The NiMaNu Project was approved by the National Ethical Committee in Niamey (Niger: 005/2013/CCNE) and the Institutional Review Board of the University of California, Davis (USA) (IRB, University of CA, Davis: 447971). Consent materials were presented in both written and oral format, in the presence of a neutral witness. Informed consent was documented with a written signature or a fingerprint prior to enrollment in the study. The study was registered at www.clinicaltrials.gov as NCT01832688.

### 2.4. Data Collection

As part of the baseline survey, each enrolled woman participated in two study visits, spaced approximately one month apart. The first visit occurred in the home, and the follow-up visit (i.e., visit 2) was conducted in a central village location. Information on socio-economic status (SES) and demographic characteristics of the woman and her household, pregnancy and health status, dietary practices, food security, and knowledge, attitudes and practices (KAP) pertaining to ANC and nutrition were collected via structured interviews by trained female fieldworkers at both study visits. Woman’s weight, height, mid-upper arm circumference (MUAC) and symphysis-fundal height (SFH) were measured in duplicate at both visits, to 50 g and 0.1 cm precision, respectively [17,18]. If the 2 measurements were >0.2 kg (weight) or >0.5 cm apart (height, MUAC, and SFH), a third measurement was taken; results represent the mean of the two closest measurements. Low MUAC was defined as <23 cm [19]. 

Dietary MN adequacy was assessed using the Minimum Dietary Diversity for Women (MDD-W) as a proxy indicator; the population of women who reported consuming at least five of ten defined food groups in the previous 24 h (using a list-based food frequency questionnaire) was considered to have a higher likelihood of MN adequacy [20]. Household SES was estimated using three proxy indices (housing quality, household assets, and household livestock), as previously described in detail elsewhere [21]. Household food insecurity, defined as the limited or uncertain access to adequate food of sufficient quality, was assessed using the Household Food Insecurity Access Scale (HFIAS) of the Food and Nutrition Technical Assistance/USAID [22]. IFA coverage was defined as the individual having received IFA supplements at any point during her current pregnancy. IFA adherence was defined as the individual having consumed IFA daily in the previous week. Gestational age was estimated as a weighted average of the following obtained information: reported last menstrual period (LMP; by estimated number of months, lunar cycles and/or proximity to a religious or cultural event), time elapsed since quickening, and two fundal height measurements taken approximately one month apart [23,24]. Trimester of pregnancy was defined as follows: 1st trimester, <13 weeks; 2nd trimester: ≥13 weeks to <27 weeks; and 3rd trimester, ≥27 weeks. 

### 2.5. Biochemical Assessments

Capillary blood samples were collected at visit 2 for the measurement of Hb in all women. In a sub-set of women, 7.5 mL venous blood samples were collected at the same time point for measurement of plasma ferritin, soluble transferrin receptor (sTfR), zinc (pZn), retinol binding protein (RBP), α-1-acid glycoprotein (AGP), C-reactive protein (CRP), vitamin B_12_, folate and histidine-rich protein II (HRP2) concentrations. According to procedures recommended by the International Zinc Nutrition Consultative Group (IZiNCG) [25,26], blood was drawn from the antecubital vein and collected in an evacuated, trace element free polyethylene tube containing lithium heparin (Sarstedt AG & Co., Numbrecht, Germany). In the field, samples were stored immediately between 4 and 8 °C until separation. Plasma was separated from heparinized blood at the Regional Hospital of Zinder ≤8 h from collection, by centrifuging at 3000 rpm for 10 min. Plasma was aliquoted in clear or amber (B_12_ and folate) microcentrifuge tubes and stored at −20 °C until shipment to the United States and Germany on high-salinity ice packs for laboratory analyses.

Hb concentration was measured immediately after capillary blood collection with a HemoCue 201+ photometer (Hemocue AB, Angelholm, Sweden). Plasma ferritin, sTfR, RBP, CRP, and AGP concentrations were measured using a combined sandwich ELISA technique and pZn was measured colormetrically (WAKO Chemicals GmbH, Neuss, Germany) at the VitMin Lab (Willstaett, Germany) [27]. All samples were measured in duplicate and the inter-assay CV for the control sample ranged from 3.0 to 9.2%. Plasma B_12_ and folate concentrations were measured by enzyme-based immunoanalysis using a Cobas(R) e41 analyzer (Roche Diagnostics, Switzerland) and the reagents vitamin B12 (theoretical normality values 95% CI: 191–663 pg/mL) and folate III (4.6–18.7 ng/mL). Plasma HRP2 concentrations (indicative of current or recent malaria parasitemia) were measured using a commercially available CELISA kit (Cellabs Pty Ltd., Brookvale, Australia), following the manufacturer’s instructions. Plasma samples were measured in singlicate on plates with positive and negative controls provided by the manufacturer; samples with an absorbance value greater than the OD of the negative control +0.1 unit were considered positive for malaria antigen.

### 2.6. Sample Size

Assuming a 50% prevalence of deficiency, the total sample size required to estimate the prevalence of each MN deficiency ±3.5% (95% CI) was *n* = 784. Venous blood for assessment of MN concentrations was collected from all eligible pregnant women participating in visit 2 of the NiMaNu Project until the required sample size was obtained. The study had a power of 80% to detect regression coefficients between continuous variables when the absolute value of the standardized coefficient was ≥0.122, assuming an intra-cluster correlation coefficient of 0.01. 

### 2.7. Statistical Analyses

Descriptive statistics were calculated for all variables. Variables not normally distributed (ferritin, sTfR, folate, B_12_) were log transformed, and characterized by parametric distribution, prior to subsequent analysis. Biomarkers considered to be acute phase proteins or reactants (ferritin, pZn, RBP) were adjusted for elevated CRP and AGP to remove the confounding effect of sub-clinical infection and inflammation on the assessment of nutritional status and deficiency. A continuous regression method was used to adjust MN biomarker concentrations to the 10th percentile of CRP and AGP concentrations for the study-specific population (CRP = 0.38 mg/L; AGP = 0.23 g/L) [28,29].

MN deficiency cut-offs in pregnant women are not well established, especially in a setting with a high prevalence of sub-clinical infection and inflammation. Therefore, the following cut-offs were used for descriptive purposes only; all analyses were conducted using continuous MN concentrations to retain the maximum information available. Anemia was defined as Hb < 11.0 g/dL in the first and third trimesters and Hb < 10.5 g/dL in the second trimester [13]. Iron deficiency was variously defined as plasma ferritin <15 µg/L (unadjusted and adjusted) and sTfR > 8.3 mg/L [27,30]. Zinc deficiency was defined as pZn < 50 µg/dL [26]. Marginal vitamin A status was defined as RBP < 1.32 µmol/L [31]. Low and marginal B_12_ concentrations were defined as plasma B_12_ < 148 pmol/L and 148–221 pmol/L, respectively. Low folate was defined as plasma folate concentration < 10 nmol/L [32,33]. Multiple micronutrient (MMN) deficiency was calculated for each woman as the sum total number of micronutrients [iron (ferritin and/or sTfR), zinc, vitamin A (RBP), B_12_ and/or folate] for which she was categorized as deficient (range = 0–5). Elevated CRP and AGP were defined as concentrations >5 mg/L and >1 g/L, respectively. 

Associations between independent risk factors and outcome variables (Hb, plasma ferritin, sTfR, pZn, RBP, B_12_, and folate) were evaluated using bivariate generalized linear mixed models (SAS PROC GLIMMIX). The association between independent risk factors and MMN deficiency was evaluated using mixed-model logistic regression (SAS PROC GLIMMIX). Hierarchical clustering models used a random effect of village nested within CSI (i.e., cluster) and a fixed effect of region. Potential predictors included SES and demographic factors, obstetric history, KAP concerning pregnancy, and current nutritional and health status. All bivariate models were reconsidered including trimester of pregnancy as an additional independent variable to assess whether any of the significant associations in the bivariate models were attributable to variation in gestational age. Although all pregnant women in the survey catchment area were eligible for participation, only one participant was in her first trimester at visit 2; therefore models controlling for trimester of pregnancy are restricted to women in their second and third trimesters. Hypothesis driven multivariable generalized linear mixed models were constructed to explore relationships between malaria antigenemia, adolescence or primigravidae, and biomarkers of anemia and iron status.

All statistical analyses were completed with SAS System software for Windows release 9.4 (SAS Institute, Cary, North Carolina, United States). Data are presented as mean (95% CI), geometric mean (95% CI), or β (95% CI) unless otherwise noted. β-coefficients from analyses using log transformed outcome variables are exponentiated regression coefficients and interpreted as percent increase/decrease (multiplicative); β coefficients from analyses using non-transformed outcome variables are interpreted as unit increase/decrease (additive). A *p* value < 0.05 was considered statistically significant. All models were evaluated for model assumptions, including normality of residuals, linearity and observations with undue leverage. 

## 3. Results

### 3.1. Participant Characteristics

**Figure 1:**
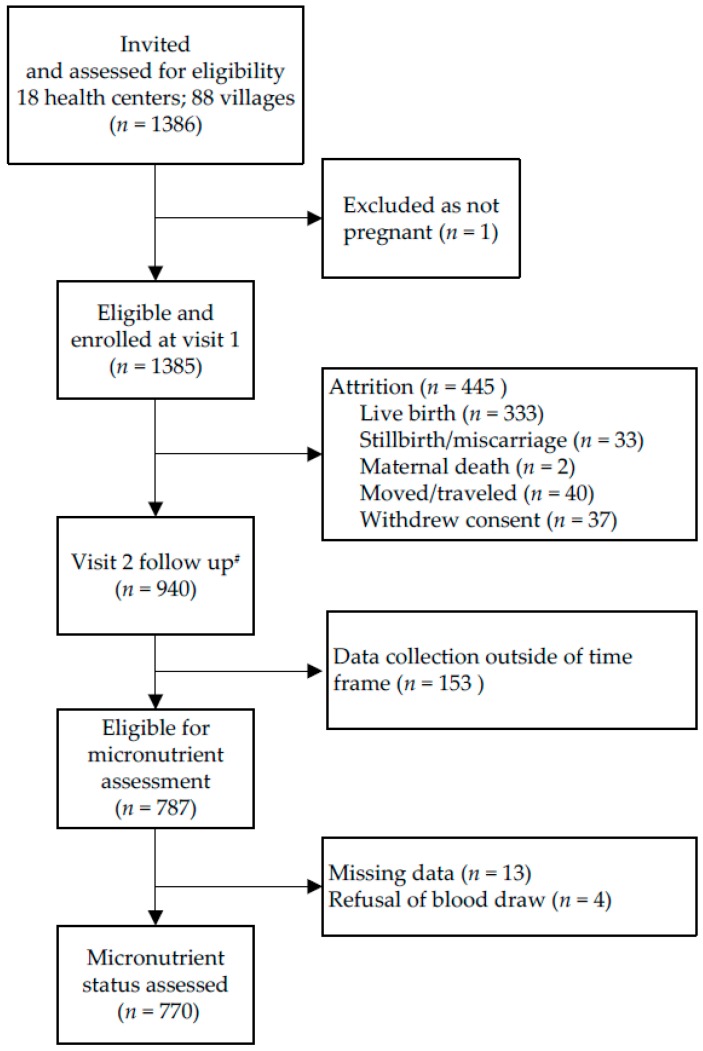
Flow chart of participant progression through the NiMaNu Project: Micronutrient status assessment.

In 88 villages, 1385 eligible pregnant women were enrolled and 940 women (67.9%) completed both visit 1 and visit 2 assessments (Figure 1); 787 of these women were eligible for biomarker assessment; and biomarkers were assessed in 770 women.

Demographic, SES and obstetric characteristics, KAP, and nutritional and health status of pregnant women eligible for biochemical assessment are presented in Table 1. Mean age at first pregnancy was 16.9 ± 2.0 years and median gravidity was 5 (IQR 4–8). In total, 53.8% of women were moderately to severely food insecure and 23.7% were undernourished (MUAC < 23.0 cm). Approximately 80% of women were anemic. Prevalence of MN deficiencies was also high, with 96.5% of women having at least one MN deficiency, and 46.5% having 3 or more co-existing MN deficiencies (Table 2).

### 3.2. Associations with Hemoglobin, Ferritin, Soluble Transferrin Receptor and Zinc

Associations between Hb, ferritin, sTfR and pZn concentrations and demographic, SES and obstetric factors, KAP, and nutritional and health status are shown in Table 3 and Table 4. Ferritin, sTfR and pZn concentrations differed significantly by trimester (Table 3); therefore, results are presented controlling for trimester to account for known physiological changes in MN concentrations during pregnancy. 

Seventy-nine percent of women were anemic. Hb concentrations were 0.3–0.7 g/dL lower among women who were adolescents (*p* < 0.01), primigravidae (*p* = 0.01), had a poor outcome in their last pregnancy (live birth, child subsequently deceased; *p* < 0.01), did not sleep under a mosquito net (*p* = 0.02), consumed clay (*p* < 0.01), or had evidence of current or recent malaria parasitemia (*p* < 0.01), compared to the reference population. Receipt of IFA supplements and reported adherence to the supplementation regimen was not associated with Hb concentration (*p* = 0.45 and *p* = 0.82, respectively). Mean Hb concentrations differed by 1.0–1.4 g/dL among seasons (*p* < 0.01) (Table 3). Hb concentrations increased significantly with increasing MUAC; a 1.0 cm increase in MUAC was associated with a 0.1 g/dL increase in Hb concentration (*p* < 0.01) Hb concentrations were positively associated with gestational weight gain; for every 1 kg/week increase in gestational weight gain, Hb concentrations were 22% higher (*p* = 0.01) (Table 4).

The overall prevalence of iron deficiency was 20.7% by ferritin (<15 µg/L); however, among women with elevated HRP2, the prevalence of ID was 10.3% (vs. 22.2% ID among women with non-elevated HRP2). Ferritin concentrations, adjusted for sub-clinical inflammation, were 21–32% higher among adolescents (*p* < 0.01), primigravidae (*p* < 0.01) and women with evidence of current or recent malaria parasitemia (*p* < 0.01). Ferritin concentrations were 22–26% lower among women who had a poor outcome in their last pregnancy (live birth, child subsequently deceased or miscarriage/stillbirth; *p* < 0.01) and 66% lower among women who consumed clay (*p* < 0.01) (Table 3), compared to the respective reference populations. As with Hb, IFA supplement coverage and adherence was not associated with ferritin concentration (*p* = 0.27 and *p* = 0.44, respectively). Ferritin concentrations were negatively associated with gestational weight gain; for every 1 kg/week increase in gestational weight gain, ferritin concentrations were 13% lower (*p* = 0.01) (Table 4). 

By sTfR (>8.3 mg/L), the prevalence of iron deficiency was 35.7%. sTfR concentrations were 9–19% lower among women who were the first wife in a polygamous marriage (*p* = 0.02), had attended primary school (*p* < 0.01), had attended ANC in their previous pregnancy (*p* < 0.01), had received IFA supplements (*p* = 0.02) and adhered to the recommended IFA supplementation regime (*p* < 0.01) and had adequate dietary diversity (*p* < 0.01) compared to the reference populations. sTfR concentrations were 21–27% higher among women had a poor outcome in their last pregnancy (live birth, child subsequently deceased or miscarriage/stillbirth; *p* < 0.01) and among women who consumed clay (*p* < 0.01). sTfR concentrations were ~30–40% lower in the hot, dry season than in the lean, rainy and post-harvest seasons (*p* = 0.04) (Table 3). sTfR concentrations were negatively associated with the housing quality and household assets index (*p* = 0.03 and *p* = 0.01, respectively) (Table 4).

The prevalence of zinc deficiency was 40.7%. pZn concentrations, adjusted for sub-clinical inflammation, were 2.6 g/dL lower among women who consumed clay compared to those who did not (*p* = 0.02) (Table 3). pZn concentrations increased significantly with increasing MUAC; a 1.0 cm increase in MUAC was associated with a 0.3 g/dL increase in pZn concentration (*p* = 0.02) (Table 4).

### 3.3. Associations with Retinol Binding Protein, Vitamin B_12_ and Folate Status

Associations between RBP, vitamin B_12_ and folate concentrations and demographic, SES and obstetric factors, KAP, and nutritional and health status are shown in Table 5 and Table 6. Plasma B_12_ and folate concentrations were significantly lower in the third compared to the second trimester; thus, all results are presented controlling for trimester to account for physiological changes in MN concentrations during pregnancy. 

Approximately 80% of women had inadequate or marginal vitamin A status. RBP concentrations, adjusted for sub-clinical inflammation, were 9–10% lower among women who were the first wife in a polygamous marriage (*p* = 0.02) and women with evidence of current or recent malaria parasitemia (*p* < 0.01) compared to the reference populations (Table 5). RBP concentrations were positively associated with food security and MUAC; a 1 unit increase in HFIAS score and a 1.0 cm increase in MUAC were associated with a 0.005 and 0.01 µmol/L increase in RBP concentration, respectively (*p* = 0.02 and *p* = 0.01) (Table 6).

The prevalence of vitamin B_12_ and folate deficiencies were 34.8% and 44.3%, respectively. Plasma B_12_ concentrations were 12% higher among women who had reportedly consumed animal source foods in the previous 24 h (*p* < 0.01) and were 7% lower among women who consumed clay (*p* < 0.01) (Table 5). B_12_ concentrations were positively associated with the housing quality index (*p* < 0.01). Plasma folate concentrations were only significantly associated with seasonality and MUAC; a 1.0 cm increase in MUAC was associated with a 1% increase in folate concentration (*p* = 0.01) (Table 6). IFA supplement coverage and adherence was not associated with plasma folate concentrations (*p* = 0.98 and *p* = 0.97, respectively).

### 3.4 Predictors of Multiple Micronutrient Deficiencies

When exploring predictors of MMN deficiencies (≥3 of the following: iron, zinc, vitamin A, vitamin B_12_ and/or folate), women in the second trimester of pregnancy were less likely to have MMN deficiencies (OR: 0.31, 95% CI: 0.21, 0.45) than women in the third trimester. Controlling for trimester of pregnancy, consumption of clay (OR: 4.88, 95% CI: 2.47, 9.66) and evidence of current or recent malaria parasitemia (OR: 2.06, 95% CI: 1.24, 3.42) were associated with an increased likelihood of MMN deficiency. The odds of having MMN deficiencies decreased with increasing age in years (OR: 0.97, 95% CI: 0.94, 1.00), increasing MUAC in cm (OR: 0.93, 95% CI: 0.87, 0.99) and increasing housing quality (OR: 0.15, 95% CI: 0.051, 0.43) (Supplementary Materials, Table S1). 

### 3.5. Multivariable Models

Primigravidae women were more likely to have evidence of current or recent malaria parasitemia than multigravidae women (OR: 0.12, 95% CI: 0.058, 0.23). Multivariable models explored relationships between primigravidae, malaria antigenemia and biomarkers of anemia and iron status (Table 7).

Overall, being primigravida versus multigravida influenced the effect of malaria antigenemia (HRP2) on Hb (*p* for interaction = 0.028) and ferritin concentrations (*p* for interaction = 0.104). Among those who did not have malaria antigenemia, there were no differences between primigravidae and multigravidae women in Hb and ferritin concentrations (*p* = 0.90 and *p* = 0.13, respectively). However, primigravidae women with recent or current malaria parasitemia (elevated HRP2) had lower Hb concentrations and higher ferritin concentrations than multigravidae women with evidence of current or recent malaria parasitemia (*p* = 0.008 and *p* = 0.004, respectively). Similar trends existed when we explored the relationships between adolescence and malaria antigenemia (HRP2) on Hb (*p* for interaction = 0.22) and ferritin concentrations (*p* for interaction = 0.09), although the effect on Hb was non-significant (data not shown).

## 4. Discussion

The present study identified a high prevalence of anemia and multiple micronutrient deficiencies among pregnant women in rural villages in the Zinder Region of Niger, which indicates the need for immediate strengthening and expansion of existing programs targeting pregnant women, including ANC services, provision of IFA supplements, intermittent prophylactic treatment of malaria and antihelminthics, and behavior change communication to promote the consumption of MN dense foods [13,14,34,35]. In addition, mass fortification of cereals, oils, and condiments (bouillon cubes) can be a useful, but perhaps not sufficient, approach to improving MN status among pregnant women. Thus, our findings justify the need for additional nutrition interventions, such as balanced protein and energy supplementation [13], targeted food fortification, and MMN supplementation. Although the recent WHO Recommendations on Antenatal Care do not support the global use of MMN supplements in pregnancy, the prevalence of MMN deficiencies in the present study population is of severe public health concern, and policymakers should consider that the benefits of MMN supplements on maternal health outcomes may outweigh the disadvantages [13,36,37]. Finally, these findings suggest that preconceptional nutrition interventions for women of reproductive age, and post-partum IFA supplementation in lactating women, may be warranted to prevent women from entering pregnancy with low nutritional status [38]. 

### 4.1. Associations between MN Deficiencies and Predictors

#### 4.1.1. Trimester

Trimester of pregnancy was a strong predictor of MN status, with women in their third trimester having significantly lower plasma ferritin, zinc, vitamin B_12_ and folate concentrations, and significantly higher sTfR concentrations, compared to women in their second trimester. Change in MN status during pregnancy is a well-established phenomenon, likely due to progressively depleted MN stores, inadequate dietary intake to meet needs for maternal and fetal tissue accumulation, and prioritization of fetal development over maternal status. However, the impact of increased plasma volume in pregnancy and increased erythropoiesis independent of MN deficiency on biomarkers of MN status is unclear, and must be considered in the interpretation of these results [7,39]. 

#### 4.1.2. Season

Season of assessment was significantly associated with anemia and sTfR concentrations, and remained significant when controlling for malaria antigenemia, food security, dietary diversity, weight gain per week and MUAC. The impact of seasonality on the remaining indicators of MN status was not significant. These results indicate that interventions are required through the calendar year, and seasonally targeted interventions are likely to be insufficient. Considering previously reported seasonal variations in food supply, food security, nutritional status and birth outcomes in this population [40] and others [41,42,43,44,45,46], we would expect season to have a significant impact on women’s MN status. However, the cluster randomization of health centers to timing of participation, and rolling implementation of this survey, meant that all health centers were not assessed at every season. Independent of the randomization matrix, all MN assessed were related to season (data not shown), but we cannot separate the effect of season and health center, which are dependent upon one another based on the randomization structure.

#### 4.1.3. Demographic and SES Factors

Adolescence during pregnancy has been shown to increase the risk of preterm birth, intra-uterine growth restriction, infant mortality and child undernutrition, and restrict young women’s linear growth [47,48]. In the present study, MN status did not differ between adolescent and adult women for the majority of MN assessed. However, adolescents did have significantly higher ferritin concentrations and significantly lower Hb concentrations than non-adolescent women; much of this association may be attributable to malaria and primigravidity. Seventy-two percent of primigravidae women were adolescents, and were more likely to have evidence of recent or current malaria parasitemia. With the exception of vitamin B_12_ status, primi- and multigravidae women whose first pregnancy occurred at a younger age did not have significantly different Hb and MN concentrations than women whose first pregnancy occurred when they were older. However, it is important to note that 90% of first pregnancies occurred during adolescence in this study population. MN status among both adolescents and adults in this population is of public health concern, and is likely to be inadequate to meet the requirements for maternal linear growth during adolescent pregnancies. Factors such as delaying first pregnancy until after adolescence, increasing birth spacing interval, and reducing the fertility rate may have positive impacts on the overall MN and health status of pregnant women in this population [49,50]. 

Indicators of economic wealth (housing quality and/or household asset index) were positively associated with plasma zinc, folate and iron status. Further demographic and SES indicators (including maternal education, ethnicity, and marital status) were not consistently associated with MN status. Thus, none of these maternal characteristics are likely to help identify women with a high risk of MN deficiencies. 

#### 4.1.4. Obstetric History

A poor outcome in their last pregnancy (child born alive who since died or miscarriage/stillbirth) was associated with lower Hb and both indicators of iron deficiency, compared to women among whom her child was born alive and is still living. Differences in household economic status (ownership index, housing quality index), ANC attendance, dietary diversity and food security did not mediate this relationship. It is possible that women who experience a poor outcome in their last pregnancy had a shorter inter-pregnancy interval, although we cannot confirm this in the present study. Some, but not all, studies have found that short inter-pregnancy and birth intervals (<6 months and <24 months, respectively) were associated with maternal anemia [51,52]. 

#### 4.1.5. Knowledge Attitudes and Practices

In the present study, 63% of women reported receiving IFA supplements during their current pregnancies, and 67% of those reported taking them daily for the past week, per WHO guidelines [13]. Although sTfR concentrations were lower with IFA supplement coverage and adherence, there were no differences in Hb, ferritin, or folate concentrations between women who had received IFA and those who had not, or between women who adhered to IFA supplementation recommendations in the week prior to the interview and those who did not. However, we only obtained reports on IFA consumption on the previous week, which may not reflect compliance during the full duration of the current pregnancy. Our findings are in contrast to a meta-analysis of daily iron supplementation finding significant impact the reduction of anemia and iron deficiency during pregnancy [6]. The lack of association between IFA supplementation and biomarkers of iron and folate status may be due to over reporting of compliance, frequent interruptions of stock at local health centers, and the fact that most women did not attend the appropriate number of ANC for their gestational age and thus not have obtained an adequate number of IFA supplements. However, this lack of association is concerning, and further underscores the need to ensure the effectiveness of the IFA supplementation program in improving iron status.

A systematic review of studies measuring dietary intake among pregnant women in sub-Saharan Africa found that mean folate, vitamin B_12_, iron and zinc were all <100% of the RNI [8]. In the present study, less than 20% of pregnant women had adequate minimum dietary diversity, which is a predictor of adequacy of MN dietary intakes [20,53]. The MDD-W is useful for population-level assessments only and does not reflect individual dietary quality because of normal day-to-day variation in dietary intakes, which may explain why MDD-W was not associated with MN status in the present study. Nevertheless, we would expect to see, and found, a high prevalence of MN deficiencies at the population level in a population with such low dietary adequacy. 

The essential nutrition actions (ENA) recommend that pregnant women consume one additional meal per day, and eat more food than usual during pregnancy [54]. In the present study, only 15–20% of pregnant women reported increasing dietary intake and frequency. Self-reported changes in the quantity of food consumed or the number of meals consumed during pregnancy compared to when the woman was not pregnant were not related to indicators of MN status, However, it has to be noted that the quantity of food reportedly consumed was based on a woman’s perception of her dietary intake, and may reflect changes in not only intake and but also perceived hunger. In addition, the quantity of food alone does not imply nutritional adequacy. Nutrition counseling and behavior change communication activities should focus on increasing both dietary diversity and total dietary intake.

Geophagy, the practice of eating earthy matter, was associated with significantly lower concentrations of Hb, ferritin, sTfR, pZn and vitamin B_12_. Previous studies in Africa have also indicated a positive association between geophagy and anemia and iron deficiency [55,56,57]. The causal pathway for geophagy is unclear and in the present cross-sectional survey, causality cannot be determined. Geophagy in pregnancy may be motivated by MN deficiency, and provide MN to women who are deficient; alternatively, geophagy may prevent MN absorption in the small intestine and have a negative effect of MN concentrations [57,58,59]. Considering the significant association between geophagy and increased risk of MN deficiencies in the study population, this association needs to be further explored and could be used to inform appropriate nutrition counseling. 

#### 4.1.6. Nutritional and Health Status

MUAC, an indicator of short-term nutritional status during pregnancy, was positively associated with Hb, pZn, RBP and folate concentrations. Similarly, gestational weight gain was positively associated with Hb concentration. Considering that a quarter of women had inadequate MUAC (<23 cm), and the majority had inadequate gestational weight gain, any nutrition interventions should target both macronutrient and micronutrient intakes. Successful interventions seem particularly important considering that maternal undernutrition during pregnancy is associated with intrauterine growth restriction of the fetus, and subsequent increased risk of neonatal death and stunting [1].

Elevated HRP2, indicating current or recent malaria parasitemia, was negatively associated with Hb and RBP concentrations, and positively associated with plasma ferritin concentrations. Thus, even after adjusting for APP, we likely underestimated prevalence of iron deficiency as assessed by ferritin concentration [60]. Given the relationships between malaria, nutritional status, and pregnancy outcomes [61,62], these results highlight the importance of integrating nutrition and malaria interventions during pregnancy, including the provision of prophylactic treatment of malaria during pregnancy, the utilization of insecticide treated mosquito bed nets and indoor residual spraying. Other infections and illnesses (e.g., enteric parasites and environmental enteric dysfunction) which were not assessed may also be associated with decreased MN absorption and/or increased dietary requirements, and are important to consider during antenatal care contacts.

### 4.2. Strengths and Weaknesses

Strengths of the current study include the sample size and the assessment of multiple indicators of MN status. In addition, this study is able to delineate factors that are associated with nutrition during gestation (demographic and SES factors, obstetric history, KAP, and nutritional and health status, seasonality), details often lacking in global estimates of MN deficiency based on population level data.

Several limitations must be considered. The population in the present study was a convenience sample from the baseline survey of the NiMaNu project, and may not be regionally or nationally representative. In addition, characterizations are limited to the second and third trimesters of pregnancy, as there were a limited number of participants in their first trimester. The study was cross-sectional, and cannot establish causation between predictor variables and biomarkers of MN status, or explore the relationships between preconception health and nutritional status and the current situation. Moreover, MN status is difficult to assess during pregnancy (due to altered nutrient metabolism, hemodilution, increased erythropoiesis, the acute phase response to pregnancy and the lack of specific and sensitive biomarkers) and cut-offs to define MN deficiencies based on functional outcomes during and after pregnancy have not been well established. Nevertheless, the present study is the first to present information on the extent of multiple micronutrient deficiencies in pregnant women in rural Niger.

## 5. Conclusions

In summary, the prevalence of multiple MN deficiencies among pregnant women is a severe public health problem in this population. There is sufficient evidence in the scientific literature to promote both the continuation of currently available, and the implementation of additional, nutritional and health interventions. In this population, poor iron status and anemia was observed in the context of a national policy for IFA supplementation among pregnant women. Continuation of this routine supplementation program, as currently implemented [15,21], is unlikely to be effective in ensuring adequate micronutrient status and improving pregnancy outcomes in this population. Considering the high prevalence of iron, zinc, vitamin A, B_12_, and folate deficiencies, and previously presented results on iodine deficiency in this study population [63], interventions to promote the strengthening of ANC, and access and adherence to nutrition and health interventions are urgently needed [64,65].

## Figures and Tables

**Table 1:** Initial Characteristics of study participants and their households ^1^.

Variable	Value
Participants (*n*)	787 ^2^
Current pregnancy, trimester	
First	1 (0.1)
Second	236 (30.3)
Third	542 (69.6)
Season	
June–September (Lean, rainy season)	277 (35.2)
October–February (Post-harvest, cool season)	286 (36.4)
March–May (Hot season)	223 (28.4)
Demographic and socio-economic characteristics	
Age (year) ^3^	26.5 (26.0, 26.9)
Adolescent	106 (13.8)
Household food insecurity access scale (HFIAS) score	5.4 (4.7, 6.1)
Marital status	
Monogamous	532 (68.0)
Polygamous, first wife	95 (12.2)
Polygamous, ≥2nd wife	155 (19.8)
Ethnicity, maternal	
Hausa	671 (85.3)
Tuareg	83 (10.6)
Other (minority)	33 (4.2)
Education, maternal	
None	458 (58.2)
Koranic schooling	171 (21.7)
Primary (1–6 years)	112 (14.2)
Secondary (7–14 years)	46 (5.8)
Obstetric history	
Primigravida	88 (11.2)
Age at first pregnancy (year)	16.9 (16.7, 17.0)
Attended ANC in last pregnancy	641 (91.8)
Result of last pregnancy	
Miscarriage, stillbirth	26 (3.7)
Live birth, child deceased	69 (9.9)
Live birth, child living	604 (86.4)
Knowledge, attitudes and practices	
Attended ANC in current pregnancy	562 (71.4)
IFA coverage	489 (62.9)
IFA adherence (consumed daily for past 7 days)	328 (42.3)
Utilization of mosquito net	626 (79.5)
Quantity of food consumed during pregnancy (compared to non-pregnant state)	
Increased	153 (19.5)
Decreased	406 (51.7)
No change	227 (28.9)
Number of meals consumed during pregnancy (compared to non-pregnant state)	
Increased	134 (17.1)
Decreased	352 (45.0)
No change	297 (37.9)
Adequate minimum dietary diversity—women (MDD-W) ^4^	147 (18.7)
Consumed vitamin A rich foods in past 24 h	680 (86.5)
Consumed animal source foods in past 24 h	295 (37.5)
Geophagy (consumed clay) during pregnancy	71 (9.0)
Nutritional and health status	
Height (cm)	158.5 (157.9, 158.9)
Mid-upper arm circumference (MUAC; cm)	24.7 (24.4, 25.0)
Gestational weight gain (kg/week)	0.24 (0.20, 0.28)
Nightblindness	9 (2.1)

^1^ ANC, antenatal care; IFA, iron and folic acid. ^2^ Sample size varies for the different covariates; birth spacing interval, ANC in last pregnancy and result of last pregnancy, *n* = 699 (excluding primigravidae women); night blindness, *n* = 427; ^3^ Mean (95 CI) and *n* (%), all such values; ^4^ MDD-W: percentage of women who reported consuming at least five of ten defined food groups in the previous 24 h.

**Table 2:** Micronutrient status and prevalence of deficiencies among study participants ^1^.

Variable	Unadjusted	Adjusted for CRP/AGP ^2^
Participants (*n*) ^3^	770	770
Hb concentration, g/dL ^4^	9.6 (9.5,9.8)	-
Anemia, Hb < 10.5 g/dL (2nd trimester) or 11.0 g/dL (1st, 3rd trimester)	601 (79.0)	-
Mild anemia (Hb 10.0–10.4 or 10.9 g/dL)	173 (22.7)	-
Moderate anemia (Hb 7.0–9.9 g/dL)	402 (52.8)	-
Severe anemia (Hb < 7.0 g/dL)	26 (3.4)	-
Ferritin, µg/L	30.5 (28.5, 32.7)	27.3 (25.8, 28.8)
Iron deficiency, ferritin < 15 µg/L	137 (17.8)	159 (20.7)
sTfR, mg/L	7.8 (7.5, 8.1)	-
Iron deficiency, sTfR > 8.3 mg/L	275 (35.7)	-
pZn, µg/dL	51.7 (51.0, 52.4)	52.3 (51.6, 53.0)
Zinc deficiency, pZn < 50 µg/dL ^5^	311 (43.4)	292 (40.7)
RBP, µmol/L	1.05 (1.03, 1.08)	1.07 (1.05, 1.10)
RBP < 0.70 µmol/L	68 (8.8)	57 (7.4)
Inadequate or marginal status, RBP < 1.32 µmol/L	622 (80.8)	614 (79.7)
B_12_, pmol/L	169.9 (165.1, 174.9)	-
Inadequate status, B_12_ < 148 pmol/L	259 (34.8)	-
Marginal status, B_12_, 148–221 pmol/L	324 (43.5)	-
Folate, nmol/L	10.5 (10.1, 10.9)	-
Inadequate status, folate < 10 nmol/L	328 (44.3)	-
Micronutrient deficiency anemia ^6^		
Iron deficiency anemia (Anemia + ferritin < 15 µg/L)	124 (16.3)	142 (18.7)
Iron deficiency anemia (Anemia + sTfR > 8.3 mg/L)	250 (32.9)	-
B_12_ deficiency anemia (Anemia + B12 < 148 pmol/L)	208 (28.3)	-
Folate-deficiency anemia (Anemia + and folate < 10 nmol/L)	265 (36.4)	-
Multiple micronutrient (MNN) deficiency ^7^		
0	25 (3.6)	24 (3.5)
1	131 (19.0)	131 (19.0)
2	212 (30.8)	222 (32.3)
3	173 (25.2)	164 (23.8)
4	114 (16.6)	114 (16.6)
5	33 (4.8)	33 (4.8)
AGP, g/L	0.39 (0.38,0.40)	-
AGP > 1 g/L	23 (3.0)	-
CRP, mg/L	2.2 (2.0, 2.4)	-
CRP > 5 mg/L	189 (24.6)	-
Malaria antigenemia (elevated HRP2) ^8^	97 (12.6)	-

^1^ AGP, α-1-acid glycoprotein; CRP, C-reactive protein; Hb, hemoglobin; sTfR, soluble transferrin receptor; RBP, retinol binding protein; pZn, plasma zinc concentration; MNN, multiple micronutrient; ^2^ Continuous adjustment to the 10th percentile of study specific CRP and AGP concentrations. ^3^ Sample size by outcome: pZn, *n* = 724; B_12_, *n* = 745; folate, *n* = 740; MMN deficiency, *n* = 688; ^4^ Mean (95% CI), geometric mean (95% CI) and *n* (%), all such values; ^5^ PZC < 55 µg/dL for first trimester (*n* = 1); ^6^ Anemia, Hb < 10.5 g/dL (2nd trimester) or 11.0 g/dL (1st, 3rd trimester); ^7^ MMN deficiency: Iron (ferritin and/or sTfR), zinc, vitamin A (RBP), B_12_ and/or folate; ^8^ Elevated plasma HRP2 (histidine-rich protein 2) concentrations are indicative of current or recent malaria parasitemia.

**Table 3:** Predictors of anemia, iron and zinc status in pregnant women, controlling for trimester: linear regression analyses (dichotomous and ordinal independent variables) and ANCOVA analyses (non-ordinal, multi-categorical independent variables) ^1^.

Variable	Hemoglobin (g/dL)		Ferritin, Adjusted for APP (µg/L) ^2,3^		Soluble Transferrin Receptor (mg/L) ^2^		Zinc, Adjusted for APP (µg/dL) ^3^	
	Mean (95% CI)	*p*	Geometric Mean (95% CI)	*p*	Geometric Mean (95% CI)	*p*	Mean (95% CI)	*p*
Current pregnancy, trimester ^4^		0.64		<0.01		<0.01		<0.01
First	NA		NA		NA		NA	
Second	9.7 (9.5, 9.9)		32.0 (29.2, 35.1)		7.0 (6.6, 7.5)		55.5 (54.3, 56.7)	
Third	9.6 (9.5, 9.8)		25.4 (23.8, 27.0)		8.1 (7.8, 8.5)		50.9 (50.1, 51.7)	
Season		<0.01		0.14		0.04		0.29
June–September (Lean, rainy season)	9.5 (9.1, 9.9)	0.01	24.0 (20.1, 28.8)	0.15	8.1 (7.2, 9.2)	0.04	54.0 (51.7, 56.2)	0.71
October–February (Post-harvest, cool season)	9.1 (8.6, 9.5)	<0.01	25.5 (20.5, 31.6)	0.05	8.8 (7.7, 10.2)	<0.01	50.9 (48.2, 53.7)	0.37
March–May (Hot season)	10.5 (10.0, 11.1)	REF	34.4 (26.7, 44.3)	REF	6.3 (5.3, 7.5)	REF	51.9 (48.7, 55.1)	REF
Demographic and socio-economic characteristics								
Adolescent		<0.01		<0.01		0.50		0.64
Yes	9.2 (8.9, 9.5)		32.1 (28.0, 36.8)		8.0 (7.3, 8.7)		52.7 (51.0, 54.4)	
No	9.7 (9.6, 9.8)		26.5 (25.0, 28.1)		7.7 (7.4, 8.1)		52.3 (51.6, 53.0)	
Marital status		0.25		0.95		0.02		0.79
Monogamous	9.7 (9.5, 9.8)	REF	27.1 (25.4, 28.8)	REF	7.8 (7.4, 8.1)	REF	52.3 (51.5, 53.1)	REF
Polygamous, first wife	9.6 (9.3, 9.9)	0.55	27.7 (24.0, 32.0)	0.76	7.0 (6.4, 7.7)	0.04	52.0 (50.2, 53.8)	0.78
Polygamous, ≥2nd wife	9.5 (9.2, 9.7)	0.10	27.1 (24.2, 30.3)	0.99	8.2 (7.7, 8.9)	0.13	52.7 (51.3, 54.2)	0.57
Ethnicity, matrnal		0.17		0.05		0.05		0.25
Hausa	9.7 (9.5, 9.8)	REF	28.0 (26.5, 29.6)	REF	7.6 (7.3, 8.0)	REF	52.5 (51.8, 53.2)	REF
Tuareg	9.5 (9.1, 9.8)	0.36	23.1 (19.6, 27.2)	0.03	8.4 (7.6, 9.4)	0.09	50.6 (48.5, 52.7)	0.10
Other (minority)	9.2 (8.7, 9.7)	0.09	23.8 (18.6, 30.5)	0.21	8.9 (7.6, 10.4)	0.05	52.4 (49.4, 55.5)	0.95
Education, maternal		0.91		0.19		<0.01		0.20
None	9.6 (9.5, 9.8)	REF	26.8 (25.0, 28.7)	REF	8.0 (7.6, 8.4)	REF	51.9 (51.0, 52.8)	REF
Koranic schooling	9.6 (9.4, 9.9)	0.87	26.6 (23.9, 29.6)	0.90	7.9 (7.4, 8.5)	0.76	52.6 (51.2, 54.0)	0.36
Primary (1–6 years)	9.5 (9.3, 9.8)	0.52	31.1 (27.2, 35.6)	0.05	6.9 (6.3, 7.5)	<0.01	53.9 (52.1, 55.6)	0.04
Secondary (7–14 years)	9.6 (9.2, 10.0)	0.87	25.7 (20.9, 31.5)	0.70	7.4 (6.5, 8.4)	0.26	51.8 (49.2, 54.4)	0.97
Obstetric history								
Primigravida		0.01		<0.01		0.56		0.72
Yes	9.3 (9.0, 9.6)		34.9 (30.1, 40.4)		8.0 (7.2, 8.8)		52.6 (50.8, 54.5)	
No	9.7 (9.5, 9.8)		26.4 (25.1, 27.9)		7.7 (7.4, 8.1)		52.3 (51.5, 53.0)	
Attended ANC in last pregnancy		0.63		0.35		0.01		0.11
Yes	9.7 (9.5, 9.8)		26.6 (25.1, 28.1)		7.6 (7.3, 7.9)		52.4 (51.7, 53.2)	
No	9.8 (9.4 , 10.1)		24.2 (20.1, 29.1)		9.1 (8.0, 10.2)		50.5 (48.2, 52.8)	
Result of last pregnancy, *n* (%)		<0.01		<0.01		<0.01		0.12
Miscarriage, stillbirth	9.5 (8.9, 10.0)	0.32	20.3 (15.4, 26.7)	<0.01	9.3 (7.8, 11.1)	<0.01	54.1 (50.6, 57.7)	0.24
Live birth, child deceased	9.0 (8.7, 9.4)	<0.01	21.2 (17.9, 25.0)	0.04	9.1 (8.2, 10.1)	0.02	54.0 (51.9, 56.2)	0.07
Live birth, child living	9.7 (9.6, 9.9)	REF	27.3 (25.8, 28.9)	REF	7.5 (7.3, 7.9)	REF	52.0 (51.2, 52.7)	REF
Knowledge, attitudes and practices								
Attended ANC in current pregnancy		0.88		0.15		0.31		0.19
Yes	9.6 (9.5, 9.8)		27.9 (26.3, 29.7)		7.7 (7.3, 8.0)		52.0 (51.2, 52.8)	
No	9.6 (9.4, 9.9)		25.6 (23.1, 28.3)		8.0 (7.5, 8.5)		53.0 (51.7, 54.3)	
IFA supplement coverage		0.45		0.27		0.02		0.54
Yes	9.6 (9.4, 9.8)		27.8 (26.0, 29.8)		7.5 (7.2, 7.9)		52.1 (51.3, 53.0)	
No	9.7 (9.5, 9.8)		26.0 (23.8, 28.5)		8.2 (7.7, 8.7)		52.6 (51.5, 53.8)	
IFA supplement adherence		0.82		0.44		<0.01		0.45
Yes	9.6 (9.4, 9.8)		27.8 (25.6, 30.2)		7.4 (7.0, 7.8)		52.0 (51.0, 53.0)	
No	9.6 (9.5, 9.8)		26.6 (24.8, 28.6)		8.1 (7.7, 8.5)		52.5 (51.6, 53.4)	
Utilization of mosquito net		0.02		0.46		0.29		0.52
Yes	9.7 (9.6, 9.8)		26.9 (25.4, 28.6)		7.7 (7.4, 8.0)		52.2 (51.4, 53.0)	
No	9.4 (9.1, 9.6)		28.3 (25.2, 31.8)		8.0 (7.5, 8.7)		52.7 (51.3, 54.2)	
Quantity of food consumed during pregnancy		0.99		0.05		0.42		0.29
Increased	9.6 (9.4, 9.9)	0.92	27.1 (24.2, 30.4)	0.23	7.6 (7.0, 8.1)	0.22	52.7 (51.3, 54.1)	0.20
Decreased	9.6 (9.5, 9.8)	0.94	28.7 (26.7, 30.8)	0.02	7.7 (7.3, 8.1)	0.30	52.6 (51.7, 53.5)	0.14
No change	9.6 (9.4, 9.8)	REF	24.7 (22.4, 27.3)	REF	8.0 (7.5, 8.6)	REF	51.5 (50.2, 52.7)	REF
Number of meals consumed during pregnancy		0.37		0.30		0.08		0.13
Increased	9.8 (9.5, 10.0)	0.20	27.3 (24.2, 30.9)	0.49	7.3 (6.7, 7.9)	0.02	53.1 (51.6, 54.6)	0.09
Decreased	9.6 (9.4, 9.8)	0.97	28.4 (26.3, 30.7)	0.12	7.7 (7.3, 8.1)	0.21	52.7 (51.8, 53.7)	0.08
No change	9.6 (9.4, 9.8)	REF	26.0 (23.8, 28.3)	REF	8.1 (7.6, 8.5)	REF	51.5 (50.4, 52.6)	REF
Adequate minimum dietary diversity—women (MDD-W)		0.46		0.70		<0.01		0.62
Yes	9.5 (9.3, 9.8)		27.8 (24.7, 31.3)		7.1 (6.6, 7.7)		52.6 (51.1, 54.2)	
No	9.6 (9.5, 9.8)		27.1 (25.6, 28.7)		7.9 (7.6, 8.2)		52.2 (51.5, 53.0)	
Consumed vitamin A rich foods in past 24 h		0.17		0.59		0.23		0.92
Yes	9.6 (9.5, 9.7)		27.1 (25.6, 28.6)		7.8 (7.5, 8.2)		52.3 (51.6, 53.0)	
No	9.8 (9.5, 10.1)		28.2 (24.6, 32.4)		7.4 (6.8, 8.1)		52.4 (50.6, 54.1)	
Consumed animal source foods in past 24 h		0.49		0.30		0.29		0.93
Yes	9.7 (9.5, 9.9)		26.3 (24.1, 28.6)		7.6 (7.2, 8.0)		52.3 (51.3, 53.4)	
No	9.6 (9.4, 9.8)		27.8 (26.0, 29.7)		7.9 (7.5, 8.2)		52.3 (51.4, 53.1)	
Geophagy (consumed clay)		<0.01		<0.01		<0.01		0.02
Yes	9.1 (8.7, 9.4)		17.2 (14.6, 20.4)		10.4 (9.3, 11.6)		50.0 (47.9, 52.1)	
No	9.7 (9.5, 9.8)		28.5 (27.0, 30.1)		7.5 (7.2, 7.9)		52.6 (51.8, 53.3)	
Nutritional and health status								
Malaria antigenemia (elevated HRP2)		<0.01		<0.01		0.59		0.23
Yes	9.0 (8.7, 9.3)		33.8 (29.3, 39.0)		8.0 (7.2, 8.7)		51.3 (49.5, 53.1)	
No	9.7 (9.6, 9.9)		26.5 (25.1, 27.9)		7.7 (7.4, 8.1)		52.5 (51.7, 53.2)	

^1^ APP, acute phase proteins; ANC, antenatal care; IFA, iron and folic acid; HRP2, histidine rich protein II; REF, reference; ^2^ Ferritin and sTfR: logarithmically transformed; ^3^ Ferritin and zinc: continuous adjustment to the 10th percentile of study specific C-reactive protein and α-1-acid glycoprotein concentrations; ^4^ Data exclude participants in first trimester, *n* = 1.

**Table 4:** Predictors of anemia, iron and zinc status in pregnant women, controlling for trimester: linear regression analyses (continuous variables) ^1^.

Variable	Hemoglobin		Ferritin, Adjusted for APP ^2,3^		Soluble Transferrin Receptor ^2^		Zinc, Adjusted for APP ^3^	
	β (95% CI)	*p*	β (95% CI)	*p*	β (95% CI)	*p*	β (95% CI)	*p*
Demographic and socio-economic characteristics ^4^								
Age (year)	0.026 (0.0098, 0.041)	<0.01	−0.0056 (−0.014, 0.0025)	0.18	−0.00074 (−0.0057, 0.0043)	0.77	−0.0088 (−0.11, 0.094)	0.87
Age at first pregnancy (year)	−0.0063 (−0.055, 0.043)	0.80	0.010 (−0.014, 0.036)	0.40	0.0082 (−0.0070, 0.024)	0.29	0.25 (−0.056, 0.55)	0.11
HFIAS score	−0.0069 (−0.027, 0.013)	0.49	0.0039 (−0.0058, 0.014)	0.43	0.0036 (−0.0026, 0.0098)	0.25	0.058 (−0.064, 0.18)	0.35
Housing quality index	0.60 (−0.047, 1.2)	0.07	0.19 (−0.13, 0.63)	0.27	−0.20 (−0.35, −0.027)	0.03	4.50 (0.55, 8.46)	0.03
Household ownership index	0.099 (−0.0010, 0.20)	0.05	0.0020 (−0.046, 0.053)	0.94	−0.039 (−0.068, −0.0085)	0.01	0.52 (−0.10, 1.15)	0.10
Nutritional and health status ^3^								
Mid-upper arm circumference (cm)	0.099 (0.060, 0.14)	<0.01	−0.012 (−0.031, 0.0071)	0.21	−0.011 (−0.023, 0.0015)	0.08	0.28 (0.045, 0.52)	0.02
Gestational weight gain (kg/week)	0.22 (0.0033, 0.43)	0.047	−0.13 (−0.22, −0.027)	0.01	−0.062 (−0.13, 0.0036)	0.06	−0.52 (−1.9, 0.83)	0.45

^1^ APP, acute phase proteins; HFIAS, Household Food Insecurity Access Scale; ^2^ Ferritin and sTfR: logarithmically transformed, β values are back-transformed to be interpreted as a multiplicative effect in the original unit; ^3^ Ferritin and zinc: continuous adjustment to the 10th percentile of study specific C-reactive protein and α-1-acid glycoprotein concentrations; ^4^ Data exclude participants in first trimester, *n* = 1.

**Table 5:** Predictors of vitamin A, B_12_ and folate status in pregnant women, controlling for trimester: linear regression analyses (dichotomous and ordinal independent variables) and ANCOVA analyses (non-ordinal, multi-categorical independent variables) ^1^.

Variable	RBP, Adjusted for APP (µmol/L) ^2^		Vitamin B_12_ (pmol/L) ^3^		Folate (nmol/L) ^3^	
	Mean (95% CI)	*p*	Geometric Mean (95% CI)	*p*	Geometric Mean (95% CI)	*p*
Current pregnancy, trimester ^4^		0.23		0.01		0.01
First	NA		NA		NA	
Second	1.10 (1.05, 1.14)		183.7 (175.4, 192.3)		11.4 (10.8, 12.0)	
Third	1.07 (1.04, 1.10)		163.6 (158.5, 168.9)		10.1 (9.7, 10.6)	
Season		0.81		0.66		0.07
June–September (Lean, rainy season)	1.09 (1.01, 1.18)	0.53	170.2 (155.3, 186.5)	0.63	11.3 (10.2, 12.6)	0.89
October–February (Post-harvest, cool season)	1.09 (0.99, 1.19)	0.60	162.1 (145.8, 180.3)	0.37	9.2 (8.2, 10.4)	0.09
March– May (Hot season)	1.04 (0.92, 1.16)	REF	177.9 (156.8, 201.8)	REF	11.2 (9.7, 12.8)	REF
Demographic and socio-economic characteristics						
Adolescent		0.08		0.20		0.89
Yes	1.13 (1.07, 1.19)		163.1 (152.5, 174.4)		10.6 (9.8, 11.3)	
No	1.07 (1.04, 1.10)		170.7 (165.6, 175.9)		10.5 (10.1, 10.9)	
Marital status		0.02		0.09		0.48
Monogamous	1.09 (1.06, 1.12)	REF	170.8 (165.5, 176.3)	REF	10.6 (10.1, 11.0)	REF
Polygamous, first wife	1.00 (0.93, 1.06)	0.01	158.1 (147.5, 169.4)	0.04	10.1 (9.4, 10.9)	0.26
Polygamous, ≥2nd wife	1.08 (1.03, 1.13)	0.61	172.8 (163.4, 182.8)	0.71	10.4 (9.7, 11.0)	0.51
Ethnicity, maternal		0.83		0.89		0.59
Hausa	1.08 (1.05, 1.11)	REF	169.3 (164.4, 174.3)	REF	10.5 (10.1, 11.0)	REF
Taureg	1.06 (0.98, 1.13)	0.56	169.3 (156.0, 183.6)	0.99	10.1 (9.3, 11.1)	0.40
Other (minority)	1.07 (0.96, 1.18)	0.83	174.3 (154.8, 196.3)	0.63	10.8 (9.6, 12.2)	0.62
Education, maternal		0.08		0.41		0.27
None	1.08 (1.05, 1.12)	REF	166.5 (160.9, 172.3)	REF	10.5 (10.1, 11.0)	REF
Koranic schooling	1.11 (1.06, 1.16)	0.29	173.1 (164.1, 182.6)	0.21	10.8 (10.2, 11.4)	0.46
Primary (1–6 years)	1.02 (0.96, 1.08)	0.07	173.3 (162.4, 184.9)	0.27	10.0 (9.3, 10.7)	0.13
Secondary (7–14 years)	1.03 (0.94, 1.12)	0.25	176.8 (160.3, 195.0)	0.25	10.2 (9.2, 11.2)	0.48
Obstetric history						
Primigravida		0.74		0.34		0.52
Yes	1.07 (1.00, 1.13)		164.0 (152.3, 176.5)		10.3 (9.5, 11.1)	
No	1.08 (1.05, 1.11)		170.2 (165.3, 175.1)		10.5 (10.1, 10.9)	
Attended ANC in last pregnancy		0.32		0.25		0.82
Yes	1.07 (1.05, 1.10)		169.0 (164.0, 174.2)		10.5 (10.1, 11.0)	
No	1.12 (1.03, 1.20)		178.8 (163.2, 195.9)		10.4 (9.5, 11.4)	
Result of last pregnancy, *n* (%)		0.31		0.28		0.17
Miscarriage, stillbirth	1.14 (1.02, 1.26)	0.26	189.3 (165.2,217.1)	0.11	11.2 (9.9, 12.8)	0.35
Live birth, child deceased	1.11 (1.04, 1.19)	0.26	169.7 (156.4, 184.3)	0.94	9.9 (9.1, 10.8)	0.12
Live birth, child living	1.07 (1.04, 1.10)	REF	169.2 (164.1, 174.4)	REF	10.6 (10.2, 11.0)	REF
Knowledge, attitudes and practices						
Attended ANC in current pregnancy		0.96		0.57		0.37
Yes	1.08 (1.05, 1.11)		168.6 (163.3, 174.2)		10.4 (10.0, 10.9)	
No	1.08 (1.03, 1.12)		171.5 (163.1, 180.4)		10.7 (10.1, 11.3)	
IFA coverage		0.76		0.05		0.98
Yes	1.08 (1.05, 1.11)		165.9 (160.2, 171.8)		10.5 (10.0, 11.0)	
No	1.07 (1.03, 1.11)		175.7 (167.8, 183.9)		10.5 (10.0, 11.1)	
IFA adherence		0.54		0.17		0.97
Yes	1.07 (1.03, 1.11)		165.8 (159.2, 172.8)		10.5 (10.0, 11.0)	
No	1.08 (1.05, 1.12)		172.2 (166.1, 178.5)		10.5 (10.0, 11.0)	
Utilization of mosquito net		0.59		0.26		0.76
Yes	1.08 (1.05, 1.11)		170.8 (165.6, 176.1)		10.5 (10.1, 11.0)	
No	1.06 (1.01, 1.12)		164.7 (155.6, 174.4)		10.4 (9.8, 11.1)	
Quantity of food consumed during pregnancy		0.89		0.20		0.18
Increased	1.09 (1.04, 1.14)	0.73	163.5 (154.6, 173.0)	0.07	11.0 (10.3, 11.7)	0.19
Decreased	1.07 (1.04, 1.11)	0.91	168.9 (162.9, 175.2)	0.27	10.3 (9.9, 10.8)	0.73
No change	1.08 (1.03, 1.12)	REF	174.7 (166.3, 183.5)	REF	10.5 (9.9, 11.0)	REF
Number of meals consumed during pregnancy		0.05		0.20		0.59
Increased	1.11 (1.06, 1.17)	0.03	164.3 (154.7, 174.4)	0.11	10.7 (10.1, 11.4)	0.63
Decreased	1.09 (1.06, 1.13)	0.04	167.2 (160.8, 173.8)	0.15	10.4 (9.9, 10.9)	0.56
No change	1.04 (1.00, 1.08)	REF	174.1 (166.7, 181.8)	REF	10.5 (10.0, 11.1)	REF
Adequate minimum dietary diversity—women (MDD-W)		0.27		0.38		0.62
Yes	1.05 (1.00, 1.11)		173.5 (163.7, 183.9)		10.4 (9.7, 11.0)	
No	1.08 (1.05, 1.11)		168.6 (163.6, 173.6)		10.5 (10.1, 11.0)	
Consumed vitamin A rich foods in past 24 h		0.52		0.19		0.54
Yes	1.08 (1.05, 1.11)		168.4 (163.5, 173.3)		10.5 (10.1, 11.0)	
No	1.06 (1.00, 1.12)		176.7 (165.1, 189.1)		10.3 (9.6, 11.1)	
Consumed animal source foods in past 24 h		0.91		0.01		0.32
Yes	1.08 (1.04, 1.12)		177.1 (169.7, 184.8)		10.3 (9.8, 10.9)	
No	1.08 (1.04, 1.11)		165.4 (160.0, 170.9)		10.6 (10.1, 11.1)	
Consumed clay		0.10		0.01		0.64
Yes	1.02 (0.94, 1.09)		153.1 (140.7, 166.6)		10.3 (9.5, 11.2)	
No	1.08 (1.05, 1.11)		171.2 (166.3, 176.1)		10.5 (10.1, 10.9)	
Nutritional and health status						
Malaria antigenemia (elevated HRP2)		0.01		0.77		0.64
Yes	1.00 (0.93, 1.06)		167.9 (156.4, 180.2)		10.3 (9.6, 11.1)	
No	1.09 (1.06, 1.12)		169.7 (164.9, 174.7)		10.5 (10.1, 10.9)	

^1^ RBP, retinol binding protein; APP, acute phase proteins; ANC, antenatal care; IFA, iron and folic acid; HRP2, histidine rich protein 2; REF = reference; ^2^ RBP: continuous adjustment to the 10th percentile of study specific C-reactive protein and α-1-acid glycoprotein concentrations.; ^3^ Vitamin B_12_ and folate: logarithmically transformed; ^4^ Data exclude participants in first trimester, *n* = 1.

**Table 6:** Predictors of vitamin A, B_12_ and folate status in pregnant women, controlling for trimester: linear regression analyses (continuous variables) ^1^.

Variable	RBP, Adjusted for APP ^2^		Vitamin B_12_^3^		Folate ^3^	
	β (95% CI)	*p*	β (95% CI)	*p*	β (95% CI)	*p*
Demographic and socio-economic characteristics ^4^						
Age (year)	−0.0017 (−0.0052, 0.0019)	0.36	0.00035 (−0.0036, 0.0043)	0.86	0.0030 (−0.00075, 0.0020)	0.12
Age at first pregnancy (year)	−0.0059 (−0.017, 0.0048)	0.28	0.014 (0.0022, 0.026)	0.02	−0.0050 (−0.016, 0.0064)	0.39
HFIAS score	0.0049 (0.00065, 0.0092)	0.02	−0.0043 (−0.0090, 0.00035)	0.07	−0.0028 (−0.0075, 0.0020)	0.25
Housing quality index	0.045 (−0.096, 0.19)	0.53	0.28 (0.10, 0.49)	0.01	0.0025 (−0.14, 0.17)	0.98
Household ownership index	0.0096 (−0.012, 0.032)	0.39	0.020 (−0.0047, 0.044)	0.11	−0.0076 (−0.031, 0.016)	0.53
Nutritional and health status ^3^						
Mid-upper arm circumference (cm)	0.012 (0.0032, 0.020)	0.01	−0.0051 (−0.014, 0.0043)	0.29	0.012 (0.0023, 0.021)	0.01
Gestational weight gain (kg/week)	−0.039 (−0.086, 0.0084)	0.11	−0.036 (−0.085, 0.015)	0.16	0.028 (−0.023, 0.081)	0.29

^1^ RBP, retinol binding protein; APP, acute phase proteins; HFIAS, Household Food Insecurity Access Scale; ^2^ RBP: continuous adjustment to the 10th percentile of study specific C-reactive protein and α-1-acid glycoprotein concentrations; ^3^ Vitamin B_12_ and folate: logarithmically transformed, β values are back-transformed to be interpreted as a multiplicative effect in the original unit; ^4^ Data exclude participants in first trimester, *n* = 1.

**Table 7:** Relationship between primigravidae, malaria antigenemia and biomarkers of anemia and iron status ^1^.

	Malaria Antigenemia (HRP2 Elevated)		No Malaria Antigenemia (HRP2 Non-Elevated)		*p* for Interaction
	Primigravida	Multigravida	Primigravida	Multigravida	
Participants ^2^, *n*	31	66	56	616	
Hemoglobin (g/dL) ^3^	8.5 (8.0, 9.0) ^a^	9.3 (8.9, 9.6) ^b^	9.7 (9.3, 10.1) ^b,c^	9.7 (9.6, 9.9) ^c^	0.028
Ferritin, adjusted for APP (µg/L) ^4^	45.4 (35.6, 58.0) ^a^	29.4 (24.8, 34.9) ^b^	30.3 (25.2, 36.3) ^b^	26.2 (24.8, 27.6) ^b^	0.104

^1^ APP, acute phase proteins; HRP2, histidine rich protein II; ^2^ Data exclude participants in first trimester, *n* = 1; ^3^ Hemoglobin: mean (95% CI); ^4^ Ferritin: geometric mean (95% CI), continuous adjustment to the 10th percentile of study specific C-reactive protein and α-1-acid glycoprotein concentrations.

## Supplementary Materials

The following are available online at www.mdpi.com/2072-6643/9/5/430/s1, Table S1: Predictors of multiple micronutrient deficiency in pregnant women, controlling for trimester.

## Abbreviations

AGP	α-1-acid glycoprotein
ANC	antenatal care
APP	acute phase proteins
CRP	C-reactive protein
CS	health post
CSI	integrated health center
DHS	demographic and health survey
ENA	essential nutrition actions
Hb	Hemoglobin
HFIAS	Household Food Insecurity Access Scale
HRP2	histidine-rich protein II
IFA	iron and folic acid
KAP	knowledge, attitudes and practices
LMP	last menstrual period
MDD-W	minimum dietary diversity—women
MN	micronutrient
MMN	multiple micronutrient
MUAC	mid-upper arm circumference
pZn	plasma zinc
RBP	retinol binding protein
SES	socio-economic status
SFH	symphysis-fundal height
sTfR	soluble transferrin receptor
WHO	World Health Organization
