# Supplementary material for: Micronutrient Status among Pregnant Women in Zinder, Niger and Risk Factors Associated with Deficiency
Source: Nutrients. 2017 Apr 26;9(5):430. doi: 10.3390/nu9050430 (PMC5452160; doi:10.3390/nu9050430)
Supplement: Supplementary file 1 [file nutrients-09-00430-s001.docx]

**Supplemental Table 1.** Predictors of multiple micronutrient deficiency in pregnant women, controlling for trimester^1^

| VARIABLE | OR (95% CI) | *P* |
| --- | --- | --- |
| Current pregnancy, trimester^2^ |  |  |
| Second | 0.31 (0.21, 0.45) | <0.01 |
| Third | Reference |  |
| Season |  | 0.11 |
| June – September (Lean, rainy season) | 0.81 (0.26, 2.59) | 0.73 |
| October – February (Post-harvest, cool season) | 2.60 (0.65, 10.34) | 0.18 |
| March – May (Hot season) |  | REF |
| Demographic and socio-economic characteristics |  |  |
| Age (y) | 0.97 (0.94, 1.00) | 0.03 |
| Adolescent | 1.43 (0.9, 2.31) | 0.14 |
| HFIAS score | 1.00 (0.97, 1.03) | 0.96 |
| Housing quality index | 0.15 (0.051, 0.43) | <0.01 |
| Household ownership index | 0.93 (0.79, 1.09) | 0.36 |
| Marital status |  | 0.55 |
| Monogamous | Reference |  |
| Polygamous, first wife | 1.31 (0.81, 2.15) | 0.27 |
| Polygamous, > 2^nd^ wife | 1.05 (0.69, 1.59) | 0.83 |
| Ethnicity, maternal |  | 0.12 |
| Hausa | Reference |  |
| Taureg | 1.82 (1.01, 3.26) | 0.04 |
| Other (minority) | 1.25 (0.56, 2.80) | 0.58 |
| Education, maternal |  | 0.83 |
| None | Reference |  |
| Koranic schooling | 0.92 (0.61, 1.40) | 0.71 |
| Primary (1-6 y) | 0.81 (0.49, 1.34) | 0.41 |
| Secondary (7-14 y) | 1.09 (0.54, 2.19) | 0.82 |
| Obstetric history |  |  |
| Primigravida | 1.37 (0.82, 2.27) | 0.23 |
| Attended antenatal care in current pregnancy | 0.80 (0.53, 1.20) | 0.28 |
| Result of last pregnancy, n (%) |  | 0.89 |
| Miscarriage, stillbirth | 1.22 (0.47, 3.16) | 0.68 |
| Live birth, child deceased | 1.09 (0.60, 1.97) | 0.77 |
| Live birth, child living | Reference |  |
| Knowledge, attitudes and practices |  |  |
| IFA coverage | 0.69 (0.47, 1.02) | 0.06 |
| IFA adherence | 0.82 (0.57, 1.18) | 0.28 |
| Utilization of bednet | 0.96 (0.63, 1.48) | 0.87 |
| Quantity of food consumed during pregnancy |  | 0.37 |
| Increase | 0.79 (0.49, 1.29) | 0.35 |
| Decrease | 1.08 (.72, 1.61) | 0.71 |
| Same | Reference |  |
| Number of meals per day consumed during pregnancy |  | 0.26 |
| Increase | 0.74 (0.46, 1.21) | 0.24 |
| Decrease | 1.10 (0.75, 1.59) | 0.63 |
| Same | Reference |  |
| Adequate minimum dietary diversity – women (MDD-W) | 0.70 (0.46, 1.09) | 0.11 |
| Consumed Vitamin A rich foods in past 24 hours | 0.88 (0.54, 1.44) | 0.62 |
| Consumed animal source foods in past 24 hours | 0.84 (0.59, 1.20) | 0.34 |
| Consumed clay | 4.88 (2.47, 9.66) | <0.01 |
| Nutritional and health status |  |  |
| Mid-upper arm circumference (MUAC; cm) | 0.93 (0.87, 0.99) | 0.03 |
| Gestational weight gain (kg/wk) | 0.82 (0.58, 1.17) | 0.28 |
| Evidence of malaria antigenemia (elevated HRP2) | 2.06 (1.24, 3.42) | <0.01 |

^1^ Multiple micronutrient deficiency was calculated for each woman as the sum total number of micronutrients [iron (ferritin and/or sTfR), zinc, vitamin A (RBP), B_12_ and/or folate] for which she was categorized as deficient (range = 0-5). HFIAS, household food insecurity access score; HRP2, histidine rich protein II; IFA, iron and folic acid supplement; MDD-W, minimum dietary diversity, women; MUAC, mid upper arm circumference; ^2^Data exclude participants in first trimester, n = 1
